# Diagnosis of Syphilitic Bilateral Papillitis Mimicking Papilloedema 

**DOI:** 10.3201/eid2601.191122

**Published:** 2020-01

**Authors:** Alicia Gonzalez-Martinez, Sonia Quintas, Diego Celdrán Vivancos, José Cebrián, José Vivancos

**Affiliations:** Hospital Universitario de la Princesa, Madrid, Spain

**Keywords:** bilateral visual loss, eye impairment, idiopathic intracranial hypertension, optic disc swelling, papillitis, papilloedema, peripapillary retinitis, sexually transmitted infections, syphilis, bacteria, Treponema pallidum

## Abstract

Syphilis produces myriad nonspecific signs and symptoms. For example, optic disk swelling might be seen in patients with syphilis as a result of cranial hypertension (papilloedema), inflammatory optic neuritis with papillitis, or optic perineuritis. We report a case involving differential diagnosis of syphilitic bilateral papillitis mimicking papilloedema.

Syphilis, caused by infection with the bacterium *Treponema pallidum*, is a sexually transmitted infection for which incidence has been increasing since 2002, especially among adult men >55 years of age who engage in risky sex (*1*). Syphilis has earned its nickname, the “great masquerader,” because it produces myriad nonspecific signs and symptoms that make it difficult to distinguish from certain other diseases. Eye impairment occurs in >3% of cases (*2*,*3*) and can be the first manifestation (*4*). Optic nerve involvement, either unilateral or bilateral, in the form of papilloedema, perineuritis, or optic neuritis, is the second most common type of syphilitic ocular impairment (*5*). Each of these conditions shares findings from fundoscopy testing with unilateral or bilateral optic disk swelling (Table), but the etiology and, therefore, the diagnostic algorithm are different. Semiology and ophthalmological findings are the key to achieving a correct syndromic diagnosis. 

**Table:** Differential diagnosis of syphilitic optic disk swelling*

Differential diagnosis	Clinical presentation	Visual acuity	Visual fields	Optic disk appearance	Other ocular abnormalities	Lumbar CSF opening pressure†	Orbital MRI
Papilloedema	Headache, nausea, tinnitus, diplopia, neck stiffness, photophobia	Normal to slow reduction (months)	Enlarged blind spot	Swollen	Flame hemorrhages	High	Normal/flattening of the posterior sclera, dilation of the ONS, and protrusion of the optic disk head
Perineuritis	Asymptomatic	Normal	Enlarged blind spot, constricted peripheral visual field	Slightly swollen	None	Normal	ONS and orbital fat expansion and enhancement
Anterior optic neuritis (papillitis)	Ocular pain, dyschromatopsia	Reduced (hours-days)	Enlarged blind spot, central scotomas, and other field abnormalities	Swollen	None/cellular activity in the posterior vitreous, patchy diffuse retinitis	Normal	Optic nerve gadolinium enhancement

*CSF cerebrospinal fluid; MRI, magnetic resonance imaging; ONS optic nerve sheath.  †Normal lumbar CSF pressure: <25 cm H_2_O, <28 cm H_2_O in obese patients.

We describe the case of a 62-year-old man who was admitted to the neurology department at Hospital Universitario de la Princesa in Madrid, Spain, with a 4-day history of bilateral decreased visual acuity. He was obese, an active smoker, and dyslipidemic. He reported neither ocular pain nor dyschromatopsia suggestive of optic neuritis, nor headache or diplopia usually associated with intracranial hypertension. He had no known history of syphilis. Visual acuity was 20/32 in the right eye and 20/63 in the left. Pupils were equal and reactive to light, with no relative afferent pupillary defect, which is typical of unilateral optic neuritis. Slit lamp examination results were normal, showing no inflamed cells in the anterior chambers or vitreous. Neurologic examination was normal. Opening pressure of the cerebrospinal fluid (CSF) on lumbar puncture was 27 cm H_2_O, above the reference range of 5–20 cm H_2_O. The CSF white cell count was 0, with normal glucose and protein levels. A fundus examination revealed bilateral optic disk swelling and peripapillary retinitis; visual field testing revealed bilateral central scotoma and an enlarged blind spot (Appendix). 

Doctors initiated acetazolamide for suspected idiopathic intracranial hypertension (IIH), but visual acuity decreased to 20/40 in the right eye and 20/200 in the left. The rapid decrease in visual acuity and the lack of response to acetazolamide suggested optic nerve involvement, which seemed atypical for IIH in the absence of other cranial nerve impairment (being the sixth cranial nerve, which is most likely to be affected by IIH in the first place). 

A cerebral magnetic resonance imaging scan with gadolinium did not reveal any structural lesion or indirect findings of IIH. For this reason, the diagnostic study was expanded. Optical coherence tomography of the nerve fiber layer showed an increase in average thickness in both eyes, reflecting optic nerve edema. Results from laboratory tests for complete blood count, urea, electrolytes, enzymes, hormones, antinuclear antibodies, and protein electrophoresis were within normal ranges. Test results were negative for HIV. A treponemal test reacted negatively to a nontreponemal Venereal Disease Research Laboratory (VDRL) test in serum, but a VDRL test in CSF was reactive without dilution. Treatment with intravenous penicillin G (4 × 10^6^ U, every 8 h for 14 d) was initiated. Fundoscopy results were normal, visual acuity remained stable but unimproved (0/40 in the right eye and 20/200 in the left eye, which was not unexpected), and visual fields remained stable 3 months after penicillin treatment was begun (Appendix). Lumbar puncture at that time revealed normal opening pressure and negative results for VDRL test scores. 

Papilloedema refers to optic disk edema caused by increased intracranial pressure. The initial pressure of 27 cm H_2_O for this patient, despite being high, was lower than the 28 cm H_2_O in obese patients required to diagnose IIH, according to the latest International Classification of Headache Disorders standards (*6*). Syphilitic optic perineuritis is usually asymptomatic (*7*) because the inflammation is restricted to the optic nerve sheath, rather than the nerve itself (*8*). Clinical suspicion of that condition arises in the presence of optic disk swelling with normal CSF pressure and visual acuity (*9*). Optic neuritis, unlike these other conditions, is usually accompanied by rapid visual failure, as seen in this patient. Ocular pain is a common finding but not necessary for diagnosis. 

Because the optic nerve and retina are considered extensions of the central nervous system, patients with ocular syphilis should undergo lumbar puncture and CSF analysis to confirm neurologic involvement. Diagnosing neurosyphilis relies on a combination of symptoms and signs verified by laboratory studies of blood and CSF (*10*), ocular symptoms due to bilateral papillitis, and positive treponemal test in serum and VDRL in CSF. This patient met these criteria. 

Our findings underline that IIH can only be diagnosed through systematic exclusion of alternative diagnoses and the diagnosis cannot be reached by fundoscopy findings alone. Clinical findings are key points for differentiating papilloedema and ocular perineuritis from papillitis. A complete diagnostic work-up is required to rule out all other etiologic causes of optic neuropathy and, if the diagnosis of ocular syphilis is reached, a lumbar puncture should be performed to rule out neurosyphilis. 

Appendix Patient images from investigation of diagnosis of syphilitic bilateral papillitis mimicking papilloedema. ssss
