# Supplementary material for: Diagnosis of Syphilitic Bilateral Papillitis Mimicking Papilloedema
Source: Emerg Infect Dis. 2020 Jan;26(1):171–3. doi: 10.3201/eid2601.191122 (PMC6924901; doi:10.3201/eid2601.191122)
Supplement: Appendix — Patient images from investigation of diagnosis of syphilitic bilateral papillitis mimicking papilloedema. ssss [file 19-1122-Techapp-s1.pdf]

Article DOI: https://doi.org/10.3201/eid2601.191122

# Diagnosis of Syphilitic Bilateral Papillitis Mimicking Papilloedema

## Appendix

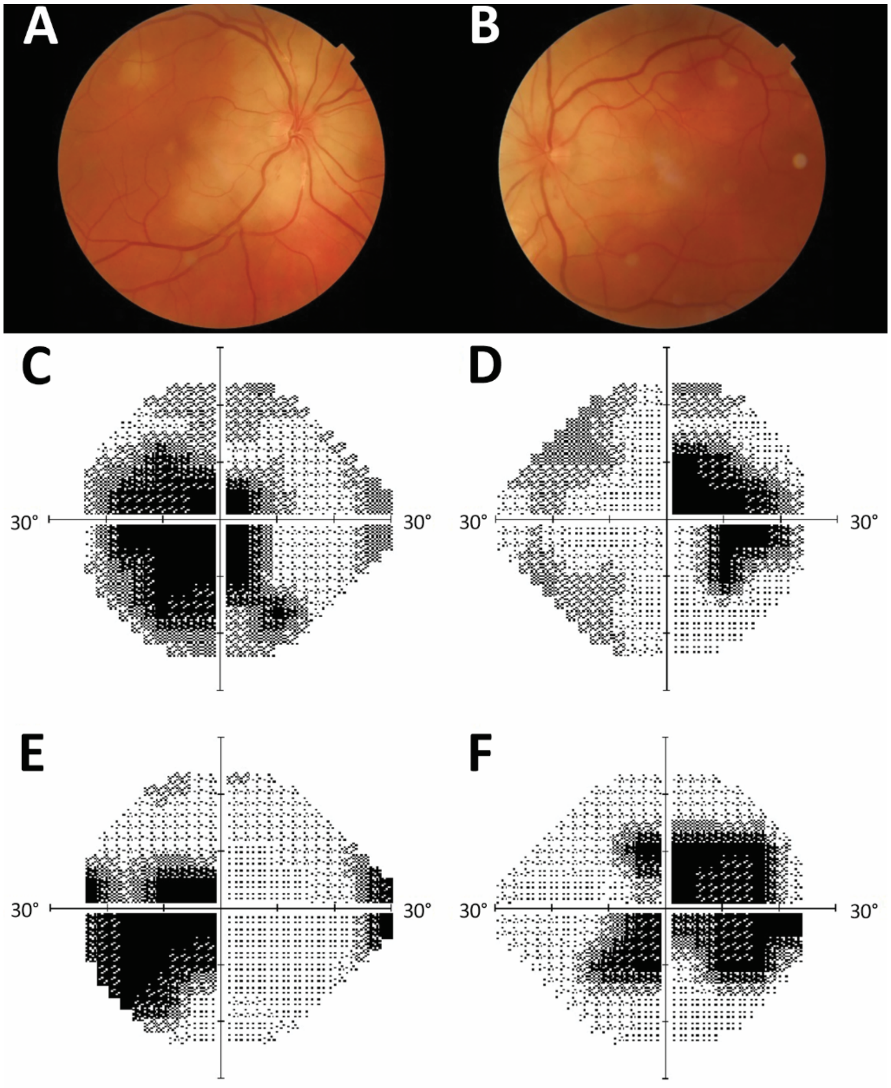


**Appendix Figure.** Patient images from investigation of diagnosis of syphilitic bilateral papillitis mimicking papilloedema. (A-B) fundoscopy showing bilateral optic disk swelling and peripapillary retinitis; (C-D) visual fields showing bilateral central scotoma and enlarged blind spot at the time of diagnosis; (E-F) visual fields after 3 months.
